# Multisystem inflammatory syndrome in children (MIS-C) secondary to COVID-19 mRNA vaccination – A case report from Qatar

**DOI:** 10.1016/j.idcr.2022.e01606

**Published:** 2022-08-18

**Authors:** Manoj Varghese, Hussam Abdel Rahman S. Alsoub, Junais Koleri, Reem Hasan Mustafa El Ajez, Ziad Mohamad Alsehli, Yaman Ismael Gh Alkailani, Muna A. Rahman S. Al Maslamani

**Affiliations:** a Division of Infectious Diseases, Hamad Medical Corporation, Qatar; b Clinical Pharmacist, Division of Infectious Diseases, Hamad Medical Corporation, Qatar; c Dept of Cardiology, Hamad Medical Corporation, Qatar; d Dept of Radiology, Hamad Medical Corporation, Qatar

**Keywords:** COVID-19, Multisystem inflammatory syndrome, MIS-C, MIS-A, COVID-19 vaccine adverse effect

## Abstract

COVID-19 vaccines are generally proven safe in all population and are highly recommended. However, rare adverse events have been reported. We hereby present a case of an 18-year-old man who presented to emergency department with fever, meningitis like symptoms, shortness of breath, chest pain, skin rash, and extreme fatigue. He had cardiac manifestations including hypotension, elevated troponin, and reduced ejection fraction. High inflammatory markers were also noted. He was initially worked up for and treated as a possible infectious etiology, but the microbiological studies were negative and there was no response to treatment. Since he had recently received booster dose of Pfizer-BioNTech COVID-19 vaccination three weeks prior to onset of symptoms, a possibility of Multisystem inflammatory syndrome in children (MIS-C) was made. His presentation fulfilled all the diagnostic criteria. The possibility for MIS-C being related to vaccination was proposed after relevant serological tests showed that the antibodies, he had were due to COVID-19 vaccine, not to a prior infection. After he received appropriate immunomodulatory treatment (IVIG and methylprednisolone) as per the guideline, he showed marked clinical improvement. Our case report highlights the need to consider MIS-C as a potential differential in young patients who present with unexplained multisystem illness with increased inflammatory markers and negative microbiologic work-up. MIS-C can be secondary to COVID-19 vaccination as well as to prior COVID-19 infection

## Introduction

As of 27th May, 2022, COVID-19 pandemic caused more than 528 million recorded cases with more than 6.2 million deaths [1]. COVID-19 infection causes a variety of disease manifestations with a spectrum of severity particularly in patients with underlying high risk factors [2]. Immune related manifestations such as cytokine release syndrome and multisystem inflammatory syndrome in children as well as adults (MIS-C and MIS-A) have also been well documented following COVID-19 infections. COVID-19 vaccines have immensely helped in controlling the epidemic and reducing mortality; however, has caused rare serious adverse effects including anaphylaxis, guillain barre syndrome, myocarditis and multisystem inflammatory syndrome [3]. We hereby present a case of a previously healthy man who presented with MIS-C secondary to COVID-19 vaccine.

## Case report

An 18-year-old male student presented to emergency department with headache, photophobia, breathlessness, and elevated troponin. He resides in Qatar with his family since many years, recently returned four days back after a one-month vacation in Jordan. His symptoms started in Jordan 3–4 days prior to arrival, as intermittent headache, fever, polyarthralgia and fatigue. He underwent a COVID-19 test in Jordan which was negative. His headache subsequently became more severe with development of photophobia, neck pain, persistent fever, and increased sleepiness. Maculopapular rashes were noted on the upper and lower extremities one day prior to admission. He was taken initially to a private clinic where he was found to have low blood pressure and elevated troponin, hence referred him to emergency department.

**Fig. 1:**
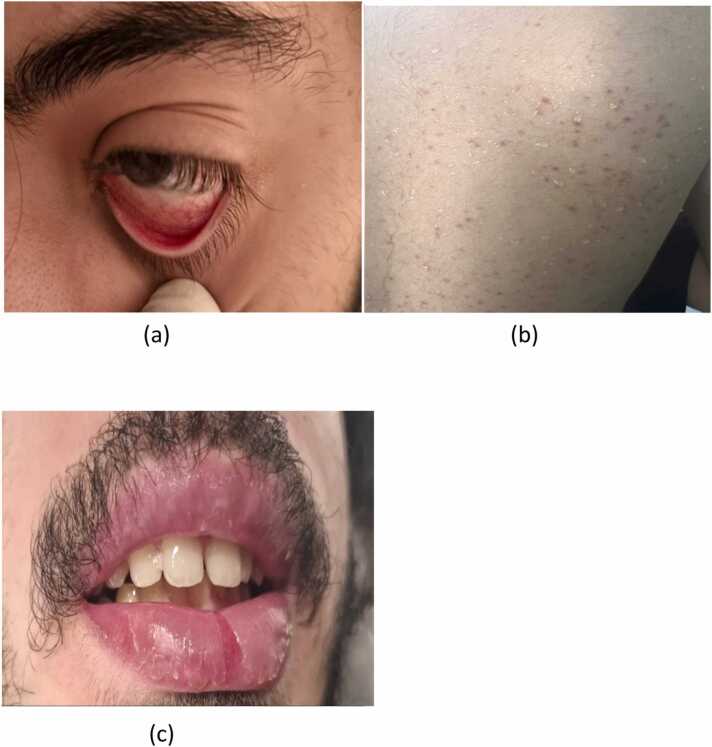
(a) Bilateral non hemorrhagic conjunctivitis. (b) Generalized erythematous maculopapular rash. (c) Oral mucositis with cracked lips.

**Fig. 2:**
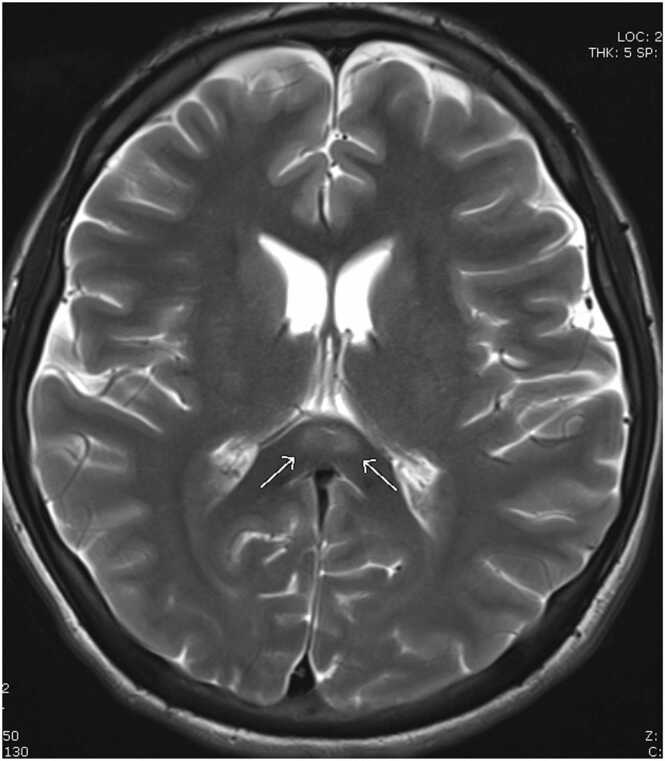
Oval shaped corpus callosum lesion with diffusion restriction, high T2 and FLAIR signal without enhancement or hemorrhagic component on SWI consistent with cytotoxic lesion of corpus callosum (Transient splenial lesion).

He denied any gastrointestinal symptoms, or any similar episodes in the past. He had no history of sick contact or tick bites while in Jordan. He received Pfizer- BioNTech COVID-19 booster dose vaccination three weeks before the onset of illness. On examination, he was febrile, tachypneic and hypotensive. Oral mucositis, bilateral non hemorrhagic conjunctivitis and generalized maculopapular rash were noted (Fig. 1a–c). Cardiac and respiratory system examination were unyielding. His laboratory parameters (Table 1) were significant for mild anemia, thrombocytopenia, elevated inflammatory markers, liver enzymes, D Dimer, and Troponins. Chest x-ray was unremarkable. Echocardiography showed mild reduced left ventricular (LV) systolic function with an ejection fraction (EF) of 45 %, mild global hypokinesis of the LV. Initial CT brain was normal. Patient was admitted with a working diagnosis of meningitis and myocarditis. Initially his family didn’t consent for lumbar puncture, hence, he was empirically started on ceftriaxone and acyclovir. MRI Brain showed cytotoxic lesion of the corpus callosum (CLOCC) involving the splenium (Fig. 2). The blood cultures and respiratory viral panel were negative including COVID-19 PCR. However, despite more than 48 hours on treatment, there was no clinical response regarding his neurological and cardiac status. At this point, Infectious Diseases team was consulted. Upon reviewing, patient fulfilled all the major, minor, and lab parameters of Brighton Collaboration Case Definition (Table 2) of Multisystem Inflammatory Syndrome in Children and Adults (MIS-C/A), hence a diagnosis of possible MIS-C post COVID-19 vaccination was made. His clinical features more suggestive of MIS-C rather than Kawasaki disease considering his age, predominantly ventricular dysfunction rather than coronary artery dilatation along with thrombocytopenia, high ferritin, and D Dimer. Cerebrospinal fluid (CSF) study was done later after convincing his parents, which ruled out meningitis. He was begun on intravenous immunoglobulin (IVIG) at a dose of 1 mg/kg/day × 2 days, IV methyl prednisolone 40 mg, 12th hourly × 3 days then oralised on discharge to prednisolone 60 mg tapered over 4 weeks and aspirin 100 mg daily. Within one day, remarkable clinical improvement was noted. His neurological symptoms and his vital signs started to stabilize, and he was back to his baseline functional status in 2 days. Advanced COVID-19 serology results suggested a positive vaccine induced immune response rather than previous infection (Spike Trimer IgG ≥ 2080 BAU/mL, Spike RBD IgM = negative, Nucleoprotein IgG = negative, Nucleoprotein IgM = negative). He was discharged on oral tapering steroids for one month. He was followed up after 2 weeks as outpatient and was asymptomatic with normalization of inflammatory markers and platelets as shown in Table 1.

**Table 1:** Relevant laboratory findings during hospitalization and at follow up at 2 weeks.

	Patient values on admission	Patient values on discharge	Patient values at 2 weeks follow-up	Normal range
**WBC (× 103/ul)**	5.5	13.5	12	4–10
**Hb (gm/dL)**	12.1	13.1	14.4	13–17
**Platelets (× 10^3^/uL)**	97	496	324	150–496
**Absolute lymphocyte count (× 10^3^/uL)**	0.8	1.7	3	1–3
**D-Dimer (mg/L FEU)**	2.13	0.89	< 0.19	0.00–0.46
**Fibrinogen (gm/L)**	7.48	–	2.14	1.7 – 4.2
**Albumin (gm/L)**	28	26	–	35–50
**ALT/AST (U/L)**	109/82	96/42	106 /30	0–40
**LDH (U/L)**	–	338	160	135–225
**Troponin T – HS (ng/L)**	364	66	9	3–15
**CRP (mg/L)**	134	28.5	< 2.0	0–5
**Procalcitonin (ng/mL)**	0.51	–	–	Less than 0.5
**Ferritin (ug/L)**	1124	751	–	20–155

Coagulation profile – Normal, Thyroid function – normal, Interleukin-6 – normal, Brucella serology – normal, blood cultures– no growth.

Nasopharyngeal swab for respiratory viral panel were all negative for: Influenza A, Parainfluenza Virus 1–4, Coronavirus (NL63,229E,OC43,HKU), hMPV, Human Bocavirus and Mycoplasma pneumoniae, Human Rhino/Enterovirus, RSV A and B, Bordetella, legionella, MERS and COVID-19.

Serology: CMV IgG and IgM – negative, EBV capsid antigen IgG and IgM – negative, Herpes Typ1 IgG– positive, herpes IgM – negative, Parvo B19 – IgG and IgM – negative.

HIV 4th generation test – negative, Hepatitis B surface Ag and Hepatitis C Ab – negative, Treponemal Antibody – negative.

CSF WBC–20, lymphocyte predominant, normal protein and sugar, CSF viral panel and VDRL – negative, CSF culture – no growth, CSF TB PCR and cultures – negative.

CSF viral panel PCR were negative for: HSV 1 and 2, Varicella Zoster virus, Parechovirus, and Enterovirus.

CSF bacterial panel were negative for (H influenza, Listeria, Neisseria, Streptococcus pneumoniae and agalactiae – negative) and CSF Cryptococcus neoformans/gatti PCR – negative.

Connective tissue and vasculitis screening – negative.

**Table 2:** Brighton collaboration case definition [4].

Age < 21 years (MIS-C) OR > 21 years (MIS-A) **AND** Fever > 3 consecutive days **AND** 2 or more of the following clinical features: • Mucocutaneous (rash, erythema, or cracking of the lips/mouth/pharynx, bilateral nonexudative conjunctivitis, erythema/edema of the hands and feet) • Gastrointestinal (abdominal pain, vomiting, diarrhea) • Shock/hypotension • Neurologic (altered mental status, headache, weakness, paresthesia’s, lethargy) **AND** Laboratory evidence of inflammation including any of the following: Elevated CRP, ESR, ferritin, or procalcitonin **AND** 2 or more measures of disease activity: • Elevated BNP or NT- proBNP or troponin • Neutrophilia, lymphopenia, or thrombocytopenia • Evidence of cardiac involvement by echocardiography or physical stigmata of heart failure • EKG changes consistent with myocarditis or myo-pericarditis **AND** Laboratory confirmed SARS-CoV-2 infection *OR* Personal history of confirmed COVID-19 within 12 weeks *OR* Close contact with known COVID-19 case within 12 weeks *OR* Following SAR-CoV-2 vaccination

## Discussion

Multisystem Inflammatory Syndrome in Children (MIS-C) was first reported in April 2020 by Riphagen et al.; in UK [5] and Verdoni et al.; in Italy [6]. Since then 8525 cases and 69 deaths due to MIS-C were reported in USA [7]. Clinical features of MIS-C are persistent fever, elevated inflammatory markers, and evidence of organ dysfunction, including myocarditis and gastrointestinal symptoms. Presentation is similar to Kawasaki syndrome, toxic shock syndrome and macrophage activation syndrome [8], [9]. MIS-C is considered as a post infectious inflammatory syndrome, given that 87 % of the patients were covid serology positive whereas only 26 % were PCR positive [10] and it responds to anti-inflammatory therapy. Moreover, a 3–4 weeks lag between peak of covid-19 cases and raise of MIS-C cases were also noted implying the timing of development of immunity [11]. Pathophysiology of MIS-C is still under investigation. Many mechanisms has been postulated [12], [13].

A case series of 15 children less than 14 years of age with MIS-C following covid-19 infection was published from Qatar [14]. All patients were admitted in hospital with five patients in intensive care unit. Patients were treated with IV immunoglobulin and corticosteroids, with anakinra in refractory cases. There was no mortality.

Our patient presented with sepsis like features including involvement of central nervous system, cardiovascular system, and gastrointestinal system with generalized rash. 3 weeks prior to the onset he received Pfizer- BioNTech booster dose. No focus of infection could be identified clinically or by imaging. Microbiological tests including viral panel, blood cultures and CSF studies were negative. There was no response to two days of empirical antibiotic treatment. At this point clinically MIS-C was considered. He was treated with IVIG, methylprednisolone and aspirin based on NHS guidelines [15]. He showed rapid response to anti-inflammatory treatment.

In a surveillance investigation from USA, Yousaf etal, identified 21 cases of MIS-C after COVID-19 vaccination [16]. Median age was 16 (range 12–20). All the patients were hospitalized with 57 % requiring intensive care. All the patients were discharged from hospital. The incidence rate was 1 case per million vaccinated individuals in this age group and in those without evidence of prior covid-19 infection, the incidence rate is 0.3 case per million population.

The first case of MIS in adult (MIS – A) post vaccination was reported in an adult female aged 44 years in United Kingdom. She presented with pain at the vaccination site followed by fever, acute kidney injury, unprovoked pulmonary embolism, elevated troponin, gastrointestinal symptoms, and rash. She responded to intravenous methyl prednisolone [17].

It is not uncommon to present with central nervous system involvement post COVID-19 or with MIS-C. There are a wide range of neurological symptoms in which most of them are transient. However, there has been rare life threatening presentation like Guillain-Barré syndrome, focal brain disorders like ischemic stroke, cerebral venous thrombosis, diffuse involvement like Acute disseminated encephalomyelitis (ADEM), severe encephalopathy with white matter and corpus callosum lesions and acute fulminant cerebral edema [18]. As seen in our case, the corpus callosum is particularly vulnerable to cytokine-induced injury due to the high density of cytokine, glutamate, and other receptors present within this region [19].

Though there are several accepted case definitions for MIS-C/A, we used the Brighton’s working group classification due to its inclusion of post SARS-CoV2 vaccination MIS-C/A [4].

Patients with Kawasaki Disease (KD) and multisystem inflammatory syndrome in children (MIS-C) could have similar presentations which can make it difficult to differentiate. However, differentiating between the two illness is important as Kawasaki disease has well defined treatment protocol. Kawasaki Disease is seen in younger patients whereas MIS-C more frequently seen in adolescents and teenagers. Coronary artery dilation or aneurysms are more common in Kawasaki Disease while elevated cardiac enzymes, ventricular dysfunction, and hemodynamic instability are seen more with MIS-C. Elevated D Dimer and hypercoagulability as well as gastrointestinal symptoms are seen more commonly in MIS-C. With regard to hematology parameters, elevated white blood cell count and eosinophilia with elevated platelets are more suggestive of the diagnosis of Kawasaki Disease, instead, patients with MIS-C more commonly develop thrombocytopenia [20].

In our case, MIS-C followed COVID-19 mRNA vaccination rather than COVID-19 infection as suggested from history as well as the serology panel. Time to onset was three weeks after the booster dose vaccination.

## Conclusion

MIS-C/MIS-A should be one of the differential diagnoses in patients who present with sepsis with multisystem involvement. It can follow COVID-19 infection and less likely, COVID-19 vaccination.

## CRediT authorship contribution statement

**Manoj Varghese:** data collection, manuscript preparation, involved in patient care. **Hussam Abdel Rahman S. Alsoub:** data collection, involved in patient care, manuscript preparation. **Junais Koleri:** manuscript preparation. **Reem Hasan Mustafa El Ajez:** Clinical pharmacist involved in patient management, manuscript preparation. **Ziad Mohamad Alsehli:** cardiologist involved in patient care, manuscript preparation. **Yaman Ismael Gh Alkailani:** manuscript preparation, provided radiology images.

## Ethical approval

Obtained from Medical Research Center, Hamad Medical Corporation.

## Consent

Written informed consent was obtained from the patient for publication of this case report and accompanying images. A copy of the written consent is available for review by the Editor-in-Chief of this journal on request.

## Conflicts of interest

No conflicts of interest.
